# {5-Chloro-2-[(4-nitro­benzyl­idene)amino]­phen­yl}(phen­yl)methanone

**DOI:** 10.1107/S1600536811046162

**Published:** 2011-11-05

**Authors:** M. Aslam, I. Anis, N. Afza, A. Nelofar, S. Yousuf

**Affiliations:** a Pharmaceutical Research Centre, PCSIR Labs. Complex, Karachi, Pakistan; b Department of Chemistry, University of Karachi, Karachi, Pakistan; c H.E.J. Research Institute of Chemistry, International Center for Chemical and Biological Sciences, University of Karachi, Karachi 75270, Pakistan

## Abstract

The mol­ecule of the title Schiff base compound, C_20_H_13_ClN_2_O_3_, assumes an *E* configuration about the C=N bond. The aromatic rings of the nitro­benzene and chloro­benzene groups are twisted by 13.89 (13)° and form dihedral angles of 76.38 (13) and 84.64 (13)°, respectively, with the phenyl ring. In the crystal, mol­ecules are linked into chains parallel to the *b* axis by C—H⋯π inter­actions.

## Related literature

For the biological activity of Schiff bases, see: Khan *et al.* (2009 ▶); Gerdemann *et al.* (2002 ▶); Samadhiya & Halve (2001 ▶); Mallikarjun & Sangamesh (1997 ▶); Fioravanti *et al.* (1995 ▶); Solomon & Lowery (1993 ▶). For related structures, see: Zeb & Yousuf (2011 ▶); Cox *et al.* (2008 ▶); Vasco-Mendez *et al.* (1996 ▶).
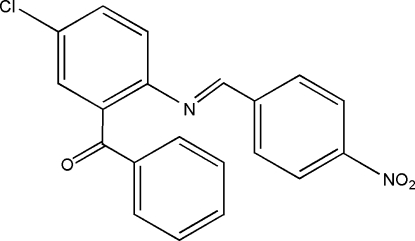


## Experimental

### 

#### Crystal data

C_20_H_13_ClN_2_O_3_
*M* *_r_* = 364.77
Monoclinic, 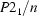

*a* = 7.231 (2) Å
*b* = 20.235 (6) Å
*c* = 11.942 (4) Å
β = 98.030 (6)°
*V* = 1730.1 (9) Å^3^
*Z* = 4
Mo *K*α radiation
μ = 0.24 mm^−1^
*T* = 273 K
0.22 × 0.13 × 0.11 mm

#### Data collection

Bruker SMART APEX CCD area-detector diffractometer
Absorption correction: multi-scan (*SADABS*; Bruker, 2000 ▶) *T*_min_ = 0.948, *T*_max_ = 0.974
9867 measured reflections
3117 independent reflections
2059 reflections with *I* > 2σ(*I*)
*R*_int_ = 0.034

#### Refinement

*R*[*F* ^2^ > 2σ(*F* ^2^)] = 0.049
*wR*(*F* ^2^) = 0.125
*S* = 1.06
3117 reflections
235 parameters
H-atom parameters constrained
Δρ_max_ = 0.17 e Å^−3^
Δρ_min_ = −0.22 e Å^−3^

### 

Data collection: *SMART* (Bruker, 2000 ▶); cell refinement: *SAINT* (Bruker, 2000 ▶); data reduction: *SAINT*; program(s) used to solve structure: *SHELXS97* (Sheldrick, 2008 ▶); program(s) used to refine structure: *SHELXL97* (Sheldrick, 2008 ▶); molecular graphics: *SHELXTL* (Sheldrick, 2008 ▶); software used to prepare material for publication: *SHELXTL*, *PARST* (Nardelli, 1995 ▶) and *PLATON* (Spek, 2009 ▶).

## Supplementary Material

Crystal structure: contains datablock(s) global, I. DOI: 10.1107/S1600536811046162/rz2660sup1.cif

Structure factors: contains datablock(s) I. DOI: 10.1107/S1600536811046162/rz2660Isup2.hkl

Supplementary material file. DOI: 10.1107/S1600536811046162/rz2660Isup3.cml

Additional supplementary materials:  crystallographic information; 3D view; checkCIF report

## Figures and Tables

**Table 1:** Hydrogen-bond geometry (Å, °) *Cg*1 is the centroid of the C1–C6 ring.

*D*—H⋯*A*	*D*—H	H⋯*A*	*D*⋯*A*	*D*—H⋯*A*
C19—H19*A*⋯*Cg*1^i^	0.93	2.68	3.538 (3)	154

Symmetry code: (i) 
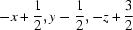
.

**Fig. 1.**
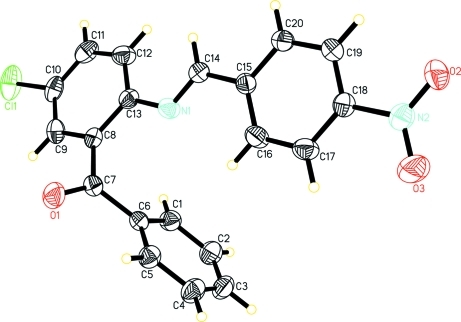
The molecular structure of the title compound with displacement ellipsoids drawn at 30% probability level.

**Fig. 2.**
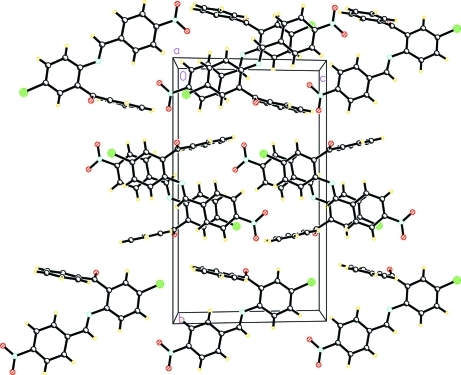
Crystal packing of the title compound viewed along the a axis.

## supplementary crystallographic information

### Comment

Schiff bases are well known reaction products of aldehyde/ketone functionalities with  amines and are considered as important ligands in coordination chemistry. They are also well known to possess a wide range of biological activities including antifungal, antiinflammatory, anti-HIV, antibacterial, herbicidal, antiproliferative, cytotoxic, anticonvulsant and anticancer activities (Khan *et al.*, 2009; Gerdemann *et al.*, 2002; Samadhiya & Halve, 2001; Mallikarjun & Sangamesh, 1997; Fioravanti *et al.*, 1995; Solomon & Lowery, 1993). The title compound was prepared and crystallized during our ongoing research on bioactive compounds.

The structure of title compound (Fig. 1) is composed of three aromatic rings *A*–*C* (C1-C6, C8-C13 and C15-C20) with dihedral angles of 76.38 (13)°, 84.64 (13)° and 13.89 (13)° between the *A*/*B*, *A*/*C* and *B*/*C* planes, respectively. The azomethine C═N double bond adopts an *E* configuration, with a C13–N1–C14–C15 torsion angle of 177.1 (2)°. The bond lengths and angles are similar to those observed in other structurally related compounds (Cox *et al.*, 2008; Vasco-Mendez *et al.*, 1996; Zeb & Yousuf, 2011). The bond length of the azomethine double bond is 1.249 (3)Å. In the crystal structure (Fig. 2), the molecules are arranged into chains parallel to the *b* axis by C—H···π interactions (Table 1).

### Experimental

The synthesis of title compound was carried out by refluxing a mixture of 4-nitrobenzaldehyde (1 mol) and 2-amino-5-chlorobenzophenone (1 mol) in ethanol (50 ml) along with 3 drops of conc. H_2_SO_4_ for 5 h at 70 °C. After cooling down to room temperature, the crystalline product was collected by filtration, washed with methanol and dried to afford the title compound in 85% yield. Recrystallization from methanol afforded yellow crystals found suitable for single-crystal X-ray diffraction studies. All chemicals were purchased from Sigma-Aldrich.

### Refinement

H atoms were positioned geometrically with C—H = 0.93 Å, and constrained to ride on their parent atoms with *U*_iso_(H) = 1.2*U*_eq_(C).

### Crystal data

**Table:**

C_20_H_13_ClN_2_O_3_	*F*(000) = 752
*M**_r_* = 364.77	*D*_x_ = 1.400 Mg m^−^^3^
Monoclinic, *P*2_1_/*n*	Mo *K*α radiation, λ = 0.71073 Å
Hall symbol: -P 2yn	Cell parameters from 1927 reflections
*a* = 7.231 (2) Å	θ = 2.7–22.4°
*b* = 20.235 (6) Å	µ = 0.24 mm^−^^1^
*c* = 11.942 (4) Å	*T* = 273 K
β = 98.030 (6)°	Block, yellow
*V* = 1730.1 (9) Å^3^	0.22 × 0.13 × 0.11 mm
*Z* = 4	

### Data collection

**Table:**

Bruker SMART APEX CCD area-detector diffractometer	3117 independent reflections
Radiation source: fine-focus sealed tube	2059 reflections with *I* > 2σ(*I*)
graphite	*R*_int_ = 0.034
ω scan	θ_max_ = 25.5°, θ_min_ = 2.0°
Absorption correction: multi-scan (*SADABS*; Bruker, 2000)	*h* = −8→8
*T*_min_ = 0.948, *T*_max_ = 0.974	*k* = −24→24
9867 measured reflections	*l* = −14→14

### Refinement

**Table:**

Refinement on *F*^2^	Primary atom site location: structure-invariant direct methods
Least-squares matrix: full	Secondary atom site location: difference Fourier map
*R*[*F*^2^ > 2σ(*F*^2^)] = 0.049	Hydrogen site location: inferred from neighbouring sites
*wR*(*F*^2^) = 0.125	H-atom parameters constrained
*S* = 1.06	*w* = 1/[σ^2^(*F*_o_^2^) + (0.0442*P*)^2^ + 0.6878*P*] where *P* = (*F*_o_^2^ + 2*F*_c_^2^)/3
3117 reflections	(Δ/σ)_max_ < 0.001
235 parameters	Δρ_max_ = 0.17 e Å^−^^3^
0 restraints	Δρ_min_ = −0.22 e Å^−^^3^

### Special details

**Table:**

Geometry. All e.s.d.'s (except the e.s.d. in the dihedral angle between two l.s. planes) are estimated using the full covariance matrix. The cell e.s.d.'s are taken into account individually in the estimation of e.s.d.'s in distances, angles and torsion angles; correlations between e.s.d.'s in cell parameters are only used when they are defined by crystal symmetry. An approximate (isotropic) treatment of cell e.s.d.'s is used for estimating e.s.d.'s involving l.s. planes.
Refinement. Refinement of *F*^2^ against ALL reflections. The weighted *R*-factor *wR* and goodness of fit *S* are based on *F*^2^, conventional *R*-factors *R* are based on *F*, with *F* set to zero for negative *F*^2^. The threshold expression of *F*^2^ > σ(*F*^2^) is used only for calculating *R*-factors(gt) *etc*. and is not relevant to the choice of reflections for refinement. *R*-factors based on *F*^2^ are statistically about twice as large as those based on *F*, and *R*- factors based on ALL data will be even larger.

### Fractional atomic coordinates and isotropic or equivalent isotropic displacement parameters (Å^2^)

**Table:**

	*x*	*y*	*z*	*U*_iso_*/*U*_eq_	
Cl1	0.19880 (13)	0.14369 (6)	0.09091 (6)	0.0991 (4)	
O1	0.5383 (3)	0.18026 (11)	0.52750 (17)	0.0748 (6)	
O2	0.2903 (3)	−0.18427 (11)	1.03849 (16)	0.0729 (6)	
O3	0.3022 (4)	−0.08484 (12)	1.09952 (18)	0.1055 (9)	
N1	0.2719 (3)	0.02909 (11)	0.54984 (17)	0.0546 (6)	
N2	0.2951 (3)	−0.12485 (13)	1.02346 (19)	0.0610 (6)	
C1	0.0800 (4)	0.16884 (13)	0.6054 (2)	0.0559 (7)	
H1B	0.0152	0.1594	0.5344	0.067*	
C2	−0.0173 (4)	0.18077 (15)	0.6959 (3)	0.0705 (9)	
H2A	−0.1472	0.1797	0.6854	0.085*	
C3	0.0798 (5)	0.19418 (15)	0.8012 (3)	0.0682 (9)	
H3A	0.0151	0.2016	0.8620	0.082*	
C4	0.2711 (5)	0.19659 (15)	0.8168 (2)	0.0631 (8)	
H4A	0.3357	0.2058	0.8879	0.076*	
C5	0.3672 (4)	0.18538 (13)	0.7273 (2)	0.0535 (7)	
H5A	0.4970	0.1875	0.7382	0.064*	
C6	0.2730 (4)	0.17095 (11)	0.6210 (2)	0.0442 (6)	
C7	0.3806 (4)	0.15890 (12)	0.5257 (2)	0.0472 (6)	
C8	0.2958 (3)	0.11941 (13)	0.42535 (19)	0.0444 (6)	
C9	0.2807 (4)	0.14706 (15)	0.3181 (2)	0.0563 (7)	
H9A	0.3118	0.1912	0.3093	0.068*	
C10	0.2197 (4)	0.10903 (17)	0.2252 (2)	0.0590 (8)	
C11	0.1768 (4)	0.04369 (17)	0.2359 (2)	0.0614 (8)	
H11A	0.1375	0.0184	0.1719	0.074*	
C12	0.1918 (4)	0.01582 (14)	0.3409 (2)	0.0610 (8)	
H12A	0.1618	−0.0285	0.3480	0.073*	
C13	0.2515 (4)	0.05276 (13)	0.43751 (19)	0.0462 (6)	
C14	0.2730 (4)	−0.03103 (13)	0.5735 (2)	0.0488 (7)	
H14A	0.2657	−0.0619	0.5154	0.059*	
C15	0.2857 (3)	−0.05421 (12)	0.6908 (2)	0.0448 (6)	
C16	0.3020 (4)	−0.01050 (14)	0.7803 (2)	0.0566 (7)	
H16A	0.3102	0.0346	0.7667	0.068*	
C17	0.3062 (4)	−0.03285 (14)	0.8895 (2)	0.0581 (8)	
H17A	0.3180	−0.0034	0.9498	0.070*	
C18	0.2927 (4)	−0.09993 (13)	0.9074 (2)	0.0478 (6)	
C19	0.2786 (4)	−0.14440 (13)	0.8209 (2)	0.0531 (7)	
H19A	0.2719	−0.1895	0.8351	0.064*	
C20	0.2745 (4)	−0.12143 (13)	0.7120 (2)	0.0528 (7)	
H20A	0.2642	−0.1512	0.6522	0.063*	

### Atomic displacement parameters (Å^2^)

**Table:**

	*U*^11^	*U*^22^	*U*^33^	*U*^12^	*U*^13^	*U*^23^
Cl1	0.0832 (7)	0.1653 (10)	0.0474 (4)	−0.0118 (6)	0.0041 (4)	0.0365 (5)
O1	0.0597 (14)	0.0966 (17)	0.0717 (14)	−0.0256 (12)	0.0214 (11)	−0.0183 (11)
O2	0.0972 (18)	0.0613 (14)	0.0608 (13)	−0.0026 (13)	0.0128 (11)	0.0164 (10)
O3	0.186 (3)	0.0809 (17)	0.0499 (13)	0.0053 (18)	0.0163 (15)	−0.0056 (12)
N1	0.0755 (18)	0.0418 (13)	0.0458 (12)	0.0014 (12)	0.0063 (11)	0.0035 (9)
N2	0.0676 (17)	0.0663 (17)	0.0484 (14)	0.0002 (14)	0.0057 (12)	0.0003 (12)
C1	0.0523 (19)	0.0556 (18)	0.0602 (17)	−0.0004 (14)	0.0091 (14)	−0.0060 (13)
C2	0.059 (2)	0.072 (2)	0.085 (2)	0.0021 (16)	0.0268 (18)	−0.0072 (17)
C3	0.089 (3)	0.063 (2)	0.0586 (19)	0.0127 (18)	0.0333 (18)	0.0035 (14)
C4	0.075 (2)	0.069 (2)	0.0452 (16)	0.0092 (17)	0.0094 (15)	0.0049 (13)
C5	0.0576 (18)	0.0553 (17)	0.0476 (15)	0.0049 (14)	0.0077 (13)	0.0037 (12)
C6	0.0493 (17)	0.0368 (14)	0.0471 (14)	−0.0004 (12)	0.0083 (12)	0.0031 (11)
C7	0.0489 (17)	0.0454 (16)	0.0477 (15)	−0.0028 (13)	0.0080 (12)	0.0039 (11)
C8	0.0408 (15)	0.0512 (16)	0.0424 (14)	0.0023 (12)	0.0108 (11)	0.0033 (11)
C9	0.0538 (18)	0.0645 (19)	0.0509 (17)	−0.0034 (15)	0.0085 (13)	0.0164 (14)
C10	0.0458 (18)	0.091 (2)	0.0409 (15)	0.0045 (16)	0.0086 (13)	0.0125 (15)
C11	0.0555 (19)	0.085 (2)	0.0417 (15)	0.0083 (17)	0.0012 (13)	−0.0088 (14)
C12	0.075 (2)	0.0539 (18)	0.0529 (17)	0.0018 (15)	0.0046 (15)	−0.0083 (13)
C13	0.0501 (17)	0.0494 (16)	0.0397 (13)	0.0052 (13)	0.0081 (12)	0.0005 (11)
C14	0.0530 (18)	0.0473 (17)	0.0476 (15)	0.0010 (13)	0.0122 (12)	−0.0015 (12)
C15	0.0436 (16)	0.0422 (15)	0.0494 (15)	0.0013 (12)	0.0098 (12)	0.0021 (11)
C16	0.071 (2)	0.0431 (16)	0.0543 (17)	−0.0057 (14)	0.0019 (14)	0.0019 (12)
C17	0.072 (2)	0.0481 (17)	0.0514 (16)	−0.0011 (15)	0.0002 (14)	−0.0074 (13)
C18	0.0462 (16)	0.0528 (17)	0.0433 (14)	0.0020 (13)	0.0027 (12)	0.0047 (12)
C19	0.0625 (19)	0.0424 (16)	0.0556 (16)	−0.0003 (13)	0.0130 (14)	0.0037 (12)
C20	0.0646 (19)	0.0447 (16)	0.0500 (15)	0.0019 (14)	0.0111 (13)	−0.0013 (12)

### Geometric parameters (Å, °)

**Table:**

Cl1—C10	1.738 (3)	C8—C13	1.398 (3)
O1—C7	1.217 (3)	C9—C10	1.371 (4)
O2—N2	1.217 (3)	C9—H9A	0.9300
O3—N2	1.213 (3)	C10—C11	1.368 (4)
N1—C14	1.249 (3)	C11—C12	1.366 (4)
N1—C13	1.413 (3)	C11—H11A	0.9300
N2—C18	1.472 (3)	C12—C13	1.392 (4)
C1—C6	1.383 (4)	C12—H12A	0.9300
C1—C2	1.390 (4)	C14—C15	1.468 (3)
C1—H1B	0.9300	C14—H14A	0.9300
C2—C3	1.379 (4)	C15—C16	1.379 (4)
C2—H2A	0.9300	C15—C20	1.388 (3)
C3—C4	1.370 (4)	C16—C17	1.377 (4)
C3—H3A	0.9300	C16—H16A	0.9300
C4—C5	1.372 (4)	C17—C18	1.380 (4)
C4—H4A	0.9300	C17—H17A	0.9300
C5—C6	1.386 (3)	C18—C19	1.364 (4)
C5—H5A	0.9300	C19—C20	1.377 (3)
C6—C7	1.485 (3)	C19—H19A	0.9300
C7—C8	1.498 (3)	C20—H20A	0.9300
C8—C9	1.388 (3)		
			
C14—N1—C13	122.8 (2)	C11—C10—Cl1	119.0 (2)
O3—N2—O2	123.3 (2)	C9—C10—Cl1	119.8 (3)
O3—N2—C18	118.0 (3)	C12—C11—C10	119.6 (3)
O2—N2—C18	118.7 (2)	C12—C11—H11A	120.2
C6—C1—C2	120.1 (3)	C10—C11—H11A	120.2
C6—C1—H1B	119.9	C11—C12—C13	121.0 (3)
C2—C1—H1B	119.9	C11—C12—H12A	119.5
C3—C2—C1	119.6 (3)	C13—C12—H12A	119.5
C3—C2—H2A	120.2	C12—C13—C8	118.7 (2)
C1—C2—H2A	120.2	C12—C13—N1	125.8 (2)
C4—C3—C2	120.5 (3)	C8—C13—N1	115.5 (2)
C4—C3—H3A	119.8	N1—C14—C15	121.7 (2)
C2—C3—H3A	119.8	N1—C14—H14A	119.2
C3—C4—C5	120.0 (3)	C15—C14—H14A	119.2
C3—C4—H4A	120.0	C16—C15—C20	119.2 (2)
C5—C4—H4A	120.0	C16—C15—C14	121.4 (2)
C4—C5—C6	120.7 (3)	C20—C15—C14	119.3 (2)
C4—C5—H5A	119.6	C17—C16—C15	120.7 (3)
C6—C5—H5A	119.6	C17—C16—H16A	119.6
C1—C6—C5	119.1 (2)	C15—C16—H16A	119.6
C1—C6—C7	121.3 (2)	C18—C17—C16	118.5 (2)
C5—C6—C7	119.6 (2)	C18—C17—H17A	120.7
O1—C7—C6	121.2 (2)	C16—C17—H17A	120.7
O1—C7—C8	118.8 (2)	C19—C18—C17	122.1 (2)
C6—C7—C8	120.0 (2)	C19—C18—N2	118.5 (2)
C9—C8—C13	119.7 (2)	C17—C18—N2	119.4 (2)
C9—C8—C7	119.6 (2)	C18—C19—C20	118.8 (3)
C13—C8—C7	120.4 (2)	C18—C19—H19A	120.6
C10—C9—C8	119.7 (3)	C20—C19—H19A	120.6
C10—C9—H9A	120.2	C19—C20—C15	120.6 (2)
C8—C9—H9A	120.2	C19—C20—H20A	119.7
C11—C10—C9	121.2 (3)	C15—C20—H20A	119.7
			
C6—C1—C2—C3	−0.5 (4)	C11—C12—C13—N1	179.9 (3)
C1—C2—C3—C4	0.8 (5)	C9—C8—C13—C12	−0.4 (4)
C2—C3—C4—C5	−0.2 (5)	C7—C8—C13—C12	−173.2 (2)
C3—C4—C5—C6	−0.6 (4)	C9—C8—C13—N1	179.7 (2)
C2—C1—C6—C5	−0.3 (4)	C7—C8—C13—N1	7.0 (3)
C2—C1—C6—C7	−179.5 (2)	C14—N1—C13—C12	14.4 (4)
C4—C5—C6—C1	0.9 (4)	C14—N1—C13—C8	−165.8 (3)
C4—C5—C6—C7	−179.9 (2)	C13—N1—C14—C15	−177.1 (2)
C1—C6—C7—O1	156.5 (3)	N1—C14—C15—C16	−1.8 (4)
C5—C6—C7—O1	−22.7 (4)	N1—C14—C15—C20	176.0 (3)
C1—C6—C7—C8	−24.0 (4)	C20—C15—C16—C17	−0.4 (4)
C5—C6—C7—C8	156.8 (2)	C14—C15—C16—C17	177.4 (2)
O1—C7—C8—C9	−57.3 (4)	C15—C16—C17—C18	−0.4 (4)
C6—C7—C8—C9	123.1 (3)	C16—C17—C18—C19	1.1 (4)
O1—C7—C8—C13	115.5 (3)	C16—C17—C18—N2	−179.4 (2)
C6—C7—C8—C13	−64.1 (3)	O3—N2—C18—C19	−177.9 (3)
C13—C8—C9—C10	1.0 (4)	O2—N2—C18—C19	2.2 (4)
C7—C8—C9—C10	173.8 (2)	O3—N2—C18—C17	2.6 (4)
C8—C9—C10—C11	−1.3 (4)	O2—N2—C18—C17	−177.2 (3)
C8—C9—C10—Cl1	179.5 (2)	C17—C18—C19—C20	−1.1 (4)
C9—C10—C11—C12	1.0 (4)	N2—C18—C19—C20	179.4 (2)
Cl1—C10—C11—C12	−179.7 (2)	C18—C19—C20—C15	0.4 (4)
C10—C11—C12—C13	−0.4 (5)	C16—C15—C20—C19	0.4 (4)
C11—C12—C13—C8	0.1 (4)	C14—C15—C20—C19	−177.5 (2)

### Hydrogen-bond geometry (Å, °)

**Table:**

Cg1 is the centroid of the C1–C6 ring.

**Table:**

*D*—H···*A*	*D*—H	H···*A*	*D*···*A*	*D*—H···*A*
C19—H19A···Cg1^i^	0.93	2.68	3.538 (3)	154

Symmetry codes: (i) −*x*+1/2, *y*−1/2, −*z*+3/2.
